# Microbiological safety of flours used in follow up for infant formulas produced in Ouagadougou, Burkina Faso

**DOI:** 10.3934/microbiol.2018.2.347

**Published:** 2018-05-18

**Authors:** Larissa Y. Waré, Augustini P. Nikièma, Jean C. Meile, Saïdou Kaboré, Angélique Fontana, Noël Durand, Didier Montet, Nicolas Barro

**Affiliations:** 1 Laboratory of Molecular Biology, Epidemiology and Surveillance of Bacteria and Viruses Transmitted by Food (LaBESTA), Center for Research in Biological Sciences, Food and Nutrition (CRSBAN), Doctoral School Sciences and Technologies, University of Ouaga I Pr JOSEPH KI-ZERBO, Burkina Faso; 2 CIRAD, UMR Qualisud, F-34398 Montpellier, France; 3 Qualisud, University of Montpellier, CIRAD, Montpellier SupAgro, University of Avignon, University of Reunion, Montpellier, France

**Keywords:** follow up infant flours, bacterial pathogenic flora, toxigenic fungal species, Burkina Faso

## Abstract

The prevalence of diarrheal diseases in children aged from 6 to 24 months in Burkina Faso is 38%. These diarrheas may be due to the consumption of contaminated weaning food. Therefore, the microbiological quality of follow up infant flours used as supplement foods is a key-point to reduce children diseases. In this study, the microbiological safety of locally-produced infant flours was investigated. One hundred and ninety-nine (199) samples were collected mainly in retails outlets and in Recovery and Nutrition Education Centers. According to the Burkina Faso regulations, microbiological analyses were carried out for Total Aerobic Mesophilic Flora (TAMF), thermotolerant coliforms, *Salmonella* spp. and yeasts/molds. The bacterial and fungal isolates were identified using phenotypic and genotypic methods and the study of the production of mycotoxins was carried out from the fungal isolates. In collected samples, the TAMF count ranged from 0 to 1.8 × 10^6^ CFU/g with a total average of 6.3 × 10^4^ CFU/g. About 2% of the samples had a microbial load exceeding the standards (10^5^ CFU/g). No *Salmonella* spp. was isolated in the final infant flours. However, the presence of *Enterobacteriaceae* (*Klebsiella* spp. *Enterobacter* spp. and *Cronobacter* spp.) was detected and molecular characterization revealed also the presence of fungal species of the genus *Aspergillus* spp., *Penicillium* spp. and *Fusarium* spp. Some of these species were found to produce aflatoxins, ochratoxin A and fumonisins, which are potential carcinogenic toxins. These results demonstrated the need for a preventive approach based on the application of Hazard Analysis Critical Control Point in the food industry to ensure food safety of infant flours in Burkina Faso.

## 1. Introduction

The agri-food sector is growing up in Burkina Faso with emphasis on the commercialisation of local food products for young children. According to the Ministry in charge of food safety in Burkina Faso [1], the need for added-value for cereal products and the generation of income for women have facilitated the development of micro-enterprises and artisanal units that do not require specific equipment. A variety of processed products is available including couscous, boiled lumps, as well as supplement formulas for children under 3 years. The nutrition survey (2016) published by the Ministry of heath, gave the prevalence of chronic malnutrition of 27.3% and that of underweight of 19.2% in Burkina Faso [2]. There are many causes of malnutrition, but one of the underlying causes is the inadequacy of average food intakes [3]. Hence, the development of high-energy infant formulas became a necessity [4],[5]. Infant flours were produced by mixing various cereals and oilseeds separately crushed and completed with sugar or salt, milk and enzymes. Then, the various intermediate treatments (sorting, drying or roasting), if poorly executed, could alter or favor the development of microorganisms in the end products. Indeed, the safety of these products is not always guaranteed because the cereals and oilseeds used to produce complementary foods could be contaminated by microorganisms and/or mycotoxins. A large number of food poisoning or food-borne diseases such as hepatitis A, typhoid fever, and diarrhea diseases affect mainly children aged from 6 months to 2 years (about 6.8 episodes per year of cholera and dysentery) [6]. This report estimated that the food-borne diseases are mainly due to pathogens, toxins and chemicals, indicating that nearly 600 million people (nearly 1 on 10 worldwide) beacame sicked after eating contaminated food. Of these, 420,000 die, including 125,000 children under five years old [7],[8]. Many pathogenic bacteria and viruses have been identified as responsible agents for these eating disorders. These are *Escherichia coli*, *Shigella* spp., *Salmonella* spp., *Staphylococcus aureus*, *Streptococcus* spp., *Vibrio cholerae*, *Bacillus cereus*, *Campylobacter* spp., *Yersinia enterocolitica*, *Clostridium perfringens* and *Listeria monocytogenes* [9]–[11]. Others have noted the presence of mold producing carcinogenic toxins such as aflatoxins in corn, millet, sorghum, peanuts, sesame, soya etc. [12],[13]. These data may explain or increase the number of malnourished children. In order to evaluate the microbiological quality and health associated risks of the formulas dedicated to young children (6–24 months old) in Ouagadougou, Total Aerobic Mesophilic Flora (TAMF), thermotolerant coliforms, yeasts/molds and isolation of *Salmonella* spp. were carried out. A molecular identification of the isolated fungal strains and a verification of their production of toxins were performed.

## 2. Materials and methods

### 2.1. Study site and sample collection

This study was conducted in Ouagadougou, capital of Burkina Faso. About 199 samples of infant flours were collected from June 2013 to December 2014 in the Recovery and Nutrition Education Centers (RNEC), semi-industrial units and artisanal units (Table 1). Approximately 300 g to 500 g samples were collected aseptically in plastic bags and transported rapidly to coolers in the laboratory of the Center for Research in Biological Sciences, Food and Nutrition of the University of Ouaga I Pr Joseph Ki-Zerbo for microbiological analysis. The genotypic analysis was carried out in the laboratory of Cirad, Qualisud UMR, Montpellier/France.

**Table 1.** Origin, composition and number of infant flours samples.

Origin of samples	Composition of infant flours	Number of samples
RNEC	Flour 1: Maize, groundnut cake, vegetable oil, salt	140
	Flour 2: Sorgho, groundnut cake, vegetable oil, salt	
	Flour 3: Millet, bean, vegetable oil, salt	
	Flour 4: Rice, monkey bread, milk, sugar	
Semi-industrial units	Flour 5: Maize, peanut, soy and/or sesame, palm oil, milk, sugar, salt, CMV and enzyme	29
	Flour 6: Sorgho or millet, peanut, soy and/or sesame, palm oil, milk, sugar, salt, CMV and enzyme	
Artisanal units	Flour 7: Maize, soy, peanut, sugar and salt	30
	Flour 8: Sorgho, peanut, sugar and salt	
	Flour 9: Rice, milk, sugar	
	Flour 10: Millet, peanut, sugar and salt	
Total	−	199

CMV: Mineral and Vitamin Supplements.

### 2.2. Microbiological analysis of infant flours

Microbiological analysis was carried out in accordance with the requirements of the International Organization for Standardization (ISO). The stock solution was obtained according to ISO 4833:2003. Ten grams (10 g) of infant flours were collected aseptically and placed in a 100 mL flask containing 90 mL of sterile Buffered Peptone Water (BPW). The mixture was homogenized with a stirrer and diluted using successive decimal.

TAMF was determined according to the AFNOR method, ISO 4833:2003. One hundred microliters (100 µL) of each dilution were used to inoculate in duplicate on Petri dishes containing Mueller Hinton medium (MH; Liofilchem Diagnostic-ITALY) and incubated in an incubator at 37 °C. Colonies were counted on the plates after 18 h to 24 h of incubation.

The thermotolerant coliforms were determined by the AFNOR method, standard V08-017:1980. The seeding of 100 µL by spreading was performed in duplicate on Petri dishes containing Eosine Methylene Blue Levine medium (L-EMB; Liofilchem Diagnostic-ITALY) and incubated at 44 °C for 24 h. Characteristic bluish colonies with a dark brown center, flattened, which occasionally have a metallic luster were counted. These characteristic colonies were transplanted onto MH agar and incubated at 37 °C for 24 h and then stored in cryotubes containing 40% glycerol at −20 °C. These cryotubes were later used for identification using API 20E kits (BioMérieux, France).

*Salmonella* were determined using AFNOR method, ISO 6579:2002. A pre-enrichment was carried out in buffered peptone water (BPW; Liofilchem Diagnostic-Italy) by incubating the decimal dilutions at 37 °C. After 18 h of incubation, 1 mL of this pre-enrichment was inoculated in 9 mL of Rapaport-Vassiladis (RVS; Liofilchem Diagnostic-Italy) and incubated at 37 °C for 24 h. One hundred microliters (100 µL) of the enriched broth were used to inoculate Petri dishes containing Hektoen Enteric Agar (HEA; Liofilchem Diagnostic-Italy) and incubated at 37 °C for 24 h. Suspended green to green-blue colonies with or without black center were transplanted onto Xylose Lysine Decarboxylase (XLD; Liofilchem Diagnostic-Italy) and incubated at 37 °C for 24 h. Then the characteristic colonies were counted.

Yeasts and molds were determined by the AFNOR method, ISO 7954:1987. Thus 100 microliters (100 µL) of each dilution were seeded in duplicate by plating on Petri dishes containing the Sabouraud + chloramphenicol 0.5 g/L medium (Sab., Liofilchem Diagnostic-Italy) and incubated at 25 °C for 3 to 5 days. Yeasts and molds were enumerated and interesting mold strains were stored in cryotubes containing 40% glycerol at −20 °C. These molds were used later to test the production of fungal toxin and to carry out the molecular characterization of the strains.

#### 2.2.1. Identification of *Enterobacteriaceae*

BioMérieux's API®20E identification galleries are test kits for identification of *Enterobacteriaceae* strains. They consist of various miniaturized biochemical tests in which the inocula have been deposited. After 24 h at 37 °C, the tests were read and the *Enterobacteriaceae* were identified using BioMérieux software.

#### 2.2.2. Characterization of fungal strains

The fungal strains conserved in glycerol were grown on Potato Dextrose Agar medium (PDA, Biokar diagnoses-Austria) at 25 °C for 7 days, in order to carry out macroscopic and microscopic identification using morphological criteria.

### 2.3. Molecular characterization of mold strains

#### 2.3.1. Extraction of mold DNA by the CTAB (Cetyltrimethyl Ammonium Bromide) method

CTAB solution consists of 20 g of CTAB, 100 mL of Tris pH 1 M, 280 mL of 5 M NaCl and 40 mL of 0.5 M EDTA (Ethylene Di-amine Tetra-acetic Acid). For working solution 40 g of polyvinylpyrrolidone and 5 mL of b-mercaptoethanol were mixed for 1 L of solution called CTAB. CTAB solution was autoclaved and the vial wrapped with aluminum foil.

Thus, the mold colony was picked on a Petri dish with 50 µL of Triton-X 100 and mixed in a 1.5 mL eppendorf tube containing 500 µL of CTAB working solution and sterile glass beads (Sigma G9772). This mixture was vortexed (Vortex Genie 2 T) for 2 min, then the tube was immersed in a water bath at 65 °C for 15 min. Then 500 µL of Chloroform: isoamyl alcohol (24:1; v/v) was added to the eppendorf tube and gently agitated before passing it to the centrifuge at a maximum speed of 6000 rpm for 5 min. Four hundred (400 µL) of the upper (aqueous) phase was transferred to a new eppendorf tube containing 233 µL of isopropanol and 32 µL NH_4_OAc 7.5 M. This mixture was centrifuged for 5 min at maximum speed. The supernatant was removed and 500 µL of cold ethanol (70%) was added to the pellet. This new mixture was centrifuged at maximum speed for 5 min. The supernatant was gently removed and the pellet was allowed to air dry. The whitish pellet was re-moistened in 20 µL of sterile pure water (Millipore) for carrying out the PCR amplification.

#### 2.3.2. DNA Amplification by PCR (Polymerase Chain Reaction)

The purified genomic DNA was amplified by PCR with primers ITS 1 and ITS 4 (the PCR reaction medium is summarized in Table 2) and then the following PCR conditions were applied: initial denaturation at 94 °C for 4 min, followed by a series of cycles including denaturation at 94 °C for 40 sec, hybridization at 58 °C for 40 sec, elongation at 72 °C for 1 min, and a final elongation cycle at 72 °C for 10 min. The PCR reactions were carried out in a Thermo Cycler (PTC-100 Peltier Thermal Cycle, MJ Research Inc., USA). The amplification of these DNA fragments will make it possible to carry out the sequencing.

**Table 2.** Reaction medium for the PCR amplification of the genomic DNA.

Reactant	Volume per well (µL)	Final concentration in the mix (50 µL)
Pure water (Eppendorf)	12.75	
Tampon PCR buffer (15 mM MgCl_2_) (Qiagen)	5	1×
MgCl_2_ 10 mM (Qiagen, Germany)	7	2.5 µM
dNTPs 10 mM by Dntp (Promega, France)	3	0.2 µM
Top Taq polymerase 5 U/µL (Promega, France)	0.25	1.25 U
ITS 1(1 µM)	10	0.2 mM
ITS4(1 µM)	10	0.2 mM
Genomic DNA extraction	2	<1 µg

Sequence of primer ITS1: TCCGTAGGTGAACCTGCGG; Sequence of primer ITS4: TCCTCCGCTTATTGATATGC.

#### 2.3.3. Sequencing and sequence analysis

Sizes of the amplified DNA fragments were controlled by electrophoresis by deposits of 5 µL on 2% agarose gel with TAE buffer (Tris-Acetate-EDTA). PCR products were conditioned and sent to GATC laboratory (Biotech, Germany) for sequencing. Sequencing data were edited manually using BioEdit software. The sequences were then compared to the databases using BLAST algorithm. The identified sequences corresponding to the high scores and e-value were chosen to assign the identification.

### 2.4. Analysis of mycotoxin production by fungal isolates

The strains identified as *Aspergillus* spp., *Penicillium* spp. and *Fusarium* spp. were transplanted onto Potato Dextrose Agar (PDA, Biokar diagnoses-Austria), and then incubated at 25 °C for 5 days.

For strains potentially OTA and aflatoxins producers, i.e. *Aspergillus* spp., *Penicillium* spp., edge of fungal biomass agars plates were put on petri dishes and placed in a 4 mL vial. Two point five mL (2.5 mL) of a methanol: formic acid mixture (25:1; v:v) was added to the biomass of *Aspergillus* spp. and *Penicillium* spp. The extracts were immersed in an ultrasonic bath for 20 min before being filtered. The filtrates obtained were collected into a glass bottle, identified by High Performance Liquid Chromatography (HPLC) and quantified by spectrofluorescence (Shimadzu RF 20A, Japan) after post column derivatization with electrochemical system (Kobra Cell™ R. Biopharm Rhône Ltd, Glasgow, UK). Fluorescence detection for AFs was set at 365 nm excitation and 435 nm emission and OTA was set at 333 nm excitation and 460 nm emission. The mobile phase A was water-methanol (55:45; v/v), 119 mg of potassium bromide and 350 µL of nitric acid and the mobile phase B was water methanol (20:80; v/v), 119 mg of potassium bromide and 350 µL of nitric acid. AFs and OTA standard solutions were used for the construction of a five-point calibration curve of peak areas versus concentration (ng/mL). The operating conditions were as follows: injection volume of 100 µL of sample and standard solutions; C18 reverse-phase HPLC column, Uptisphere type, ODS, 5 µm particle size, 5 ODB, 250 × 4.6 mm, with identical pre-column, thermostatically controlled at 40 °C; isocratic flow rate of 0.8 mL/min. Mobile phase gradient: mobile phase A: 0% (0–26 min); 65% (26–45 min); 0% (45–50 min); 41% (20–25). The detection and quantification limits on aflatoxins were 0.3 µg/kg and 1 µg/kg, respectively. The detection and quantification limits on ochratoxin A were 0.05 µg/kg and 0.1 g/kg, respectively. The contents were calculated from a calibration curve established with aflatoxins (TSL-108, Biopharm Rhône Ltd, Glasgow, UK) and ochratoxin standards (TSL-504, Biopharm Rhône Ltd, Glasgow, UK).

For fumonisin-producing strains, i.e. *Fusarium* spp., 2 mL of a methanol:water (50:50; v:v) mixture was added to the biomass of *Fusarium* spp. The extracts were immersed in an ultrasonic bath for 20 min before being filtered. The filtrates obtained were collected and derivatized with O-phthaldialdehyde (OPA) prior to analyze by HPLC and quantified by spectrofluorescence (Shimadzu RF 20A, Japan). Fluorescence detection for fumonisins was set at 335 nm excitation and 440 nm emission. The mobile phase A was: acetonitrile-acetic acid (99:1; v/v), and the mobile phase B was water acetic acid (99:1; v/v). The derivatized sample was prepared as follow: 100 µL of eluate was mixed with 100 µL of OPA. The operating conditions were as follows: injection volume of 100 µL of derivatized sample; C18 reverse-phase HPLC column, Uptisphere type, ODS, 5 µm particle size, 5 ODB, 250 × 4.6 mm, with identical pre-column, thermostatically controlled at 35 °C; isocratic flow rate of 1 mL/min. Mobile phase gradient: mobile phase A: 41% (0–9 min); 61% (9–16 min); 100% (16–20 min); 41% (20–25 min). The detection and quantification limits were 5 µg/kg and 20 µg/kg, respectively. The contents were calculated from a calibration curve established with fumonisin standard solutions (TSL-202, Biopharm Rhône Ltd, Glasgow, UK).

### 2.5. Statistical analysis

After the colonies counting on Petri dishes, the result was expressed in Colony-Forming Unit (CFU)/g of sample, according to the Moroccan standard ISO 7932 as follows:

If the boxes contain 150 to 300 colonies:

$$\text{N}=\frac{\sum \text{C}}{\text{V}\left(\text{n}1+0.1\text{n}2\right)\text{x}}\text{d}$$ (1)

N is the number of microorganisms per g of food. ∑C the sum of colonies counted on all selected boxes of two successive dilutions; V is the volume generally inoculated; n1 is the number of boxes used for the first dilution; n2 is the number of boxes used for the second dilution and d is the dilution rate corresponding to the first dilution retained.

If there are less than 15 colonies on the boxes, Y is the arithmetic mean of the colonies counted on the 2 Petri dishes.

$$N=\frac{Y}{d}$$ (2)

Y is the number of microorganisms per g and d is the dilution rate corresponding to the dilution retained.

Interpretations were made according to the 3 class plan except *Salmonella* which was interpreted according to the 2 class plan described by the Government of Canada “Lignes directrices et normes pour l'interprétation des résultats analytiques en microbiologie alimentaire”. Analytiques methodes accredited ISO/CEI 17025 by the standards council of Canada (N°131).

The data was recorded with Microsoft Excel 2007 and the ANOVA were performed with XLStat PRO version 7.5.2. Interpretation of the values was carried out according to the Fisher test, the least significant difference (LSD) with a confidence interval of 95% between the UFC/g number of food and the origin of the product.

## 3. Results and discussion

Infant flours from cereal bases are preparations in the form of porridge that are given to children from six months of age as a complement to breast milk. They must contain nutrients for the growth of young children. According to the preparation, there are several types of infant flours: flours to cook, pre-cooked flours, cooked or instant flours [14]. This study focussed on the flours to cook and instant flours. Flours to cook are a mixture of cereals and oilseed or leguminous to which milk and sugar are sometimes added. They require more or less prolonged boiling. The “cooked” or “instant flours” have an equivalent composition as the previous flours to be cooked except that they are produced according more or less complex processes and they require a simple mixture with water previously boiled to obtain the porridge. Thus 95.5% (190/199) of the samples in this study are infant flours to cook and 4.5% (9/199) are instant flours.

At the level of RNEC and artisanal units, the infant flours produced were flours to cook and were manufactured in an artisanal way (little or no industrial materials). These infant flours were composed of local cereals (rice, millet, sorghum or corn), oilseeds (groundnuts, soya or sesame), sometimes legumes (sweet potatoes), sugar, milk, oil or salt.

At the level of semi-industrial units, which are small and medium-sized enterprises with adequate industrial equipment, infant flours consist in local cereals (rice, millet, sorghum or maize), certain oilseeds (peanut, sesame), legumes (tubers), sugar, milk but also certain additives such as mineral supplements, vitamins and industrial alpha-amylolytic enzymes. These semi-industrial units produce both types of infant flours. With the exception of those produced in the RNEC, the sales of infant flours mainly takes place in supermarkets, in the Center for Health and Social Promotion (CHSP) and at the place of production.

### 3.1. Microbiological analysis of infant flours

The results of the microbiological analysis were compared with the standards reported by Gret/Orstom [15] (Table 3).

**Table 3.** Microbiological standards for infant flours.

Standards (CFU/g)	Norms	
	Flour to cook	Instant flour
Total Aerobic Mesophilic Flora (TAMF)	<10^5^	<10^4^
Thermotolerant coliforms	<100	<20
*Salmonella*	0	0
Aflatoxins	0	0
Yeasts & molds	<10^3^	Not specified

Source : Gret/Orstom 1998.

In this study, the microbiological analysis of the flours to cook revealed that the majority of the samples (92.1%) were satisfactory compared to the microbiological food safety standards for infant flours (Table 4).

**Table 4.** Microbiological results for the infant flours (flours to cook).

Samples	Three-Class Criteria	TAMF (n = 190)	Thermotolerant Coliforms (n = 190)	Yeasts & Molds (n = 190)
Flours to cook	Satisfactory	175 (92.1%)	185 (97.4%)	177 (93.2%)
	Acceptable	14 (7.4%)	3 (1.6%)	17 (8.9%)
	Unsatisfactory	1 (0.5%)	3 (1.6%)	0 (0)

The results of the microbiological analysis of the instant flours revealed no unsatisfactory sample for both TAMF, thermotolerant coliform and yeast/mold (Table 5).

**Table 5.** Microbiological results for infant flours (instant flours).

Samples	Three-Class Criteria	TAMF (n = 9)	Thermotolerant Coliforms (n = 9)	Yeasts & Molds (n = 9)
Instant flours	Satisfactory	9 (100%)	8 (88.9%)	9 (100%)
	Acceptable	0 (0)	1(11.1%)	0 (0)
	Unsatisfactory	0 (0)	0 (0)	0 (0)

As shown in Table 6 the average CFU number varied between 1.1 × 10^5^ and 3.5 × 10^4^ CFU/g according to the production site.

**Table 6.** Total occurrence of microorganisms in infant flours.

Products	Percentage of unsatisfactory samples	Total average CFU/g	Range CFU/g
Infant flours from RNEC	10.7% (15/140)	3.5 × 10^4^	0.0–2.6 × 10^5^
Infant flours from semi-industrial units	13.8% (4/29)	5.2 × 10^4^	0.0–9.8 × 10^5^
Infant flours from artisanal units	3.3% (1/30)	1.1 × 10^5^	0.0–1.9 × 10^6^

The statistical analysis indicated that there was no significant difference according to the sites of production. However, Table 6 showed differences in the average CFU numbers obtained for each sample. These results showed that the occurrence of TAMF, thermotolerant coliforms and mold yeasts was unsatisfactory at 3.3% for infant flours produced by artisanal units and 10.7% for infant flours produced by RNEC, the CFU numbers reaching 1.9 × 10^6^ and 2.6 × 10^5^ respectively. It was 13.8% for the infant flours produced by the semi-industrial units with CFU values of 9.8 × 10^5^ slightly lower than those obtained by the artisanal units and slightly higher than those obtained by the RNEC.

The number of unsatisfactory samples was lower in flours to cook but the results were unsatisfactory with regard to good manufacturing practices [16]. Indeed, the bacterial counts could reach a CFU value for TAMF of 1.9 × 10^6^ in the infant flours that were produced by artisanal units. Though educational training was organized by various sanitary authorities in these units, a permanent and effective monitoring of the good practices was lacking. The emphasis was rather on the income generated by the production of these infant flours. In semi-industrial units, where monitoring for good hygiene and manufacturing practices were assumed to be stricter, we still obtained up to 13.8% of unsatisfactory infant flour samples because of lack of good practices. Yet the most part of semi-industrial units were followed by Gret with the Nutrifaso project which aimed to improve the quality of food for the children (6 to 24 months). Several works have been carried out in order to implement good production procedures [4],[17] and to follow the specifications laid down by Gret. Although the NGO and the Ministry of Health closely supervised RNEC, the results (10.7%) obtained in this study were high. In general, the unsatisfactory microbiological results obtained in this study indicate that the infant flours were altered and/or could lead to food poisoning (diarrheas deseases) in the infants who consume them. These could be due to poor hygiene production [17].

*Salmonella* spp. is a potential source of contamination after heat treatment [18]. Its absence in all the samples analyzed in this study was similar to the results obtained by other authors [19],[20]. Presently, investigations have shown that *Cronobacter* spp. was more commonly found than *Salmonella* spp. in infant flours [21],[22].

### 3.2. Identification of strains of thermotolerant coliforms and molds

#### 3.2.1. Identification of strains of thermotolerant coliforms

Identification by Api 20E kits indicated that 60% (12/20) of the isolated strains belonged to the genus *Klebsiella* (*K. pneumoniae* and *K. planticola*), 35% (7/20) to the genus *Enterobacter* (*E. cloacae* and *E. gergoviae*) and 5% (1/20) to presumptive *Cronobacter* spp. According to the Public Health Agency of Canada the species belonging to the genera *Klebsiella* spp. and *Enterobacter* spp. are commensal bacteria in the human and animal digestive tract. Thus, they may be an indicator of fecal contamination [23]. In the same publication, it was specified that species of the genus *Klebsiella* were involved in many cases of sepsis, urinary and wound infections, notably in intensive care units and neonatal sepsis. According to FAO/WHO, strains of the genera *Cronobacter* are opportunistic pathogens [18]. The natural habitat of *Cronobacter* spp. is not well understood, however, they have been isolated from a diverse range of environments, e.g. processing plants and foods or powdered infant formula [22]. In several investigations, *Cronobacter* spp. also have been isolated from a wide range of foods other than powdered infant formula, including various types of meat, fish and fish products, herbs and spices, cereals and fermented bread [21]. *Cronobacter* spp. was implicated in outbreaks of meningitis and enteritis, especially in infant and young children. A reduction in the prevalence of *Cronobacter* has to be obtained by improving hygiene with HACCP, GMP and GHP [24].

#### 3.2.2. Identification of mold strains isolated from infant flours

One hundred and fifty (150) fungal strains were isolated from infant flours. Depending on their morphology (macroscopic and microscopic) 3 main genera (*Aspergillus* spp., *Penicillium* spp. and *Fusarium* spp.) and various other genera such as *Trichoderma* spp., *Cladosporium* spp., *Geotrichum* spp., *Mucor* and *Paecilomyces* spp. could be identified. The genus *Aspergillus* spp. was the most prevalent in infant flours with a percentage of 69.7%, followed by *Penicillium* spp. with a percentage of 10.5% and finally *Fusarium* spp. with a percentage of 3.3%. The various other genera identified accounted for 5.9%. It should also be noted that nearly 10.5% of the strains isolated could not be identified by this method. Other authors have also found an abundance of the genera *Aspergillus* spp., *Fusarium* spp. and *Penicillium* spp. in corn flour samples. The proportions were 93.3% of *Aspergillus* spp., 83.3% of *Penicillium* spp., 53.3% of *Mucorales*, 43.3% of *Cladosporium* spp., 33.3% of *Fusarium* spp. The remaining species recorded in less than 20% of the samples were *Trichoderma* spp., *Beauveria* spp., *Geotrichum* spp., *Alternaria* spp., *Paecilomyces* spp., *Acremonium* spp. and *Scopulariopsis* spp. [25].

After isolation, PCR amplification and sequencing of the 28S DNA region of fungal strains, the data were compared with the National Center for Biotechnology Information (NCBI) DNA database in order to obtain the molecular identification of isolates. Concerning the genus *Aspergillus*, the following species were identified: *A. flavus*, *A. tamarii*, *A. oryzae*, *A. sydowii* and *A. carbonarius*. The species identified for the genus *Penicillium* were: *P. citrinum* and *P. georgense*. For the genus *Fusarium* the following species was identified: *F. verticilloides*. Some of these species are known to be potential producers of mycotoxins (aflatoxins, ochratoxin A, fumonisins, T2, deoxynivalenol, zearalenone).

### 3.3. Production of mycotoxins by fungal isolates

Despite of the measures to reduce the presence of contaminants, mycotoxins have been detected in infant flours [26]. Thus, after identifying the fungal strains that were isolated from the infant formulas, the ability of the identified species to produce mycotoxins have been checked. The following mycotoxins were analysed: aflatoxins, ochratoxin A and fumonisins. Table 7 below shows the results of these analyses.

**Table 7.** Analysis of mycotoxins production by fungal isolates from infant flours.

Identified Species	Production of mycotoxin in µg/kg						
	AFG2	AFG1	AFB2	AFB1	AFs	OTA	(FB1 + FB2)
*A. flavus-1*	−	−	−	26	26	−	−
*A. flavus-2*	−	−	09	436	445	−	−
*A. flavus-3*	3	17	10	1215	1245	−	−
*A. carbonarius*	−	−	−	−	−	2604	−
*A. niger*	−	−	−	−	−	−	−
*A. tamarii*	−	−	−	−	−	−	−
*A. sydowii*	−	−	−	−	−	−	−
*F.* *verticilloides*	−	−	−	−	−	−	−

AFB1: Aflatoxin B1; AFB2: Aflatoxin B2; AFG1: Aflatoxin G1; AFG2: Aflatoxin G2; AFs: Aflatoxins (G2, G1, B2, B1); OTA: Ochratoxin A; FB1 + FB2: Fumonisins (B1 + B2).

The results shown that *A. flavus* and *A. carbonarius* were able to produce notable amounts of mycotoxins (aflatoxins and ochratoxin A respectively) (Table 7). It can be noticed that the samples of infant flours from which the producing strains originated were highly contaminated [26]. However, some species of *A. flavus*, *A. niger* and *F. verticilloides* did not produce mycotoxin in the test, suggesting that any potentially toxinogenic species does not necessarily produce toxin. Indeed, the study conducted by Alborch et al. [25] showed that *A. flavus* or *A. parasiticus* were detected in 10 of the 16 positive samples on MEA medium, but only 2 strains of *A. flavus* and one strain of *A. parasiticus* were able to produce aflatoxins. *A. niger* has also been described as an OTA producer [27]–[29] but its production is uncommon because only 1–3% of isolates produced toxins in pure culture. Similarly, *Fusarium verticillioides* isolated from the infant flours samples did not produce fumonisins, whereas fumonisins are normally synthesized mainly by *Fusarium verticillioides*. Then only a few were tested to see their ability to produce mycotoxins.

Mycotoxin-producing species in infant flours can lead children to develop serious chronic diseases such as cancer and immunosuppression [30]. Indeed, Aflatoxin chronic exposure has been associated with cancer liver, delayed growth in children [31],[32]. It has also been associated with the weakening of the immune system. Recently it has also been linked to HIV and tuberculosis (TB) [33]. At high levels concentration, exposure to aflatoxins may cause haemorrhage, edema, and even immediate death [33]. Moreover, Jayaramachandran et al. [30] indicated that aflatoxin synthesis is highest when moisture is above 13% and temperature is between 24 °C and 37 °C. This is why hot geographic regions such as Burkina Faso are the most favorable environments for aflatoxins [26]. Burkina Faso does not have regulations on mycotoxins in foods, therefore; no measures have been taken for its detection in the units (RNEC, semi-industrial units and artisanal units).

These results show that infant flours used as a food supplement are rather potentially risky foods for health. They could lead to undernutrition or chronic diseases. Adopting good agricultural practices in seed, harvest, post-harvest, and treatment could reduce mycotoxin contamination in the first place.

## 4. Conclusions

Depending on the microorganisms sought in the infant flours, high rates of contamination have been observed in artisanal units. Even though overall coliform and mold contamination levels are acceptable in infant flours, the presence of microorganisms, species or toxins can pose a great hazard to the consumer, especially for a child whose immune status is still fragile. The bacterial and fungal species present in infant flours have shown that they can either alter the food or cause acute or chronic diarrheal diseases thus weakening the child health. This could put a high risk on the actions taken so far to combat malnutrition. Thus, the danger is now known. This will involve putting in place prevention mechanisms that will reduce the microorganisms present in infant flours, especially in the case of mold toxins. Further research is thus needed to find innovative solutions to address overlooked issues of aflatoxin contamination and exposure. Finally, the collection of additional information could be useful for a number of policies makers and the development of regulations on mycotoxins in foods.
